# Blood Lead and High-Density Lipoprotein Concentrations in Relation to Human Blood Pressure: A Cross Sectional Study

**DOI:** 10.3389/fnut.2022.899780

**Published:** 2022-06-15

**Authors:** Biao Hu, Pei-yao He, Nan-nan Zhong, Zi-min Gao, Jiang-long Guo, Jun-tao Feng, Chu-qin Huang, Jun-bo Yang, Dong-lin Sun

**Affiliations:** ^1^Department of Clinical Medicine, The Second Clinical School of Guangzhou Medical University, Guangzhou, China; ^2^Department of Anesthesiology, The Second Clinical School of Guangzhou Medical University, Guangzhou, China; ^3^Department of Medical Imaging, The Second Clinical School of Guangzhou Medical University, Guangzhou, China; ^4^State Key Laboratory of Respiratory Disease, National Clinical Research Centre for Respiratory Disease, Guangzhou Institute of Respiratory Health, The First Affiliated Hospital of Guangzhou Medical University, Guangzhou, Guangdong, China; ^5^Department of Gastrointestinal Surgery, The Second Affiliated Hospital School of Medicine, Southern University of Science and Technology, Shenzhen, China; ^6^Shenzhen Third People's Hospital, Shenzhen, China; ^7^Guangzhou Medical University, Guangzhou, China

**Keywords:** blood pressure, blood lead, HDL, interaction, cross sectional study

## Abstract

**Background:**

While the relationship between blood pressure and blood lead has been studied more extensively, the effect of high-density lipoprotein (HDL) concentration on this relationship remains uncertain. Therefore, this study aimed to determine the effect of HDL concentration on the relationship between blood lead and blood pressure.

**Methods:**

The research used cross-sectional data from the 2005 to 2014 National Health and Nutrition Examination Survey (NHANES), which included 16,451 participants aged 20–60 years. Multivariable linear regression was used to evaluate the correlation among blood lead, systolic blood pressure (SBP), and diastolic blood pressure (DBP). HDL concentration was determined by low HDL concentration (≤ 49 mg/dl) and high HDL concentration (>49 mg/dl) stratified. The effect of HDL concentration was assessed by an interaction test between blood lead and SBP in multivariable linear regression.

**Results:**

In this cross-sectional research, we identified a positive correlation between blood lead and SBP, but not DBP. The relationship between blood lead and SBP was different in the group with low and high HDL concentrations (β: 0.21 95% Cl:−0.05-0.46 vs. β:0.47 95% Cl: 0.15-0.79). In addition, high HDL significantly altered the positive correlation between blood lead and SBP (*P*-value of interaction < 0.001).

**Conclusion:**

The study suggests an interaction between HDL and blood lead in elevating SBP, which may have important clinical implications.

## Introduction

Hypertension is the most common chronic non-communicable disease, second only to smoking among preventable causes of death from any cause (1), and is an important global health problem. Hypertension is also the most important risk factor for cardiovascular disease, leading to half of the coronary heart disease and about two-thirds of the cerebrovascular disease burden (2).

Lead is a relatively common environmental toxin that can cause many acute and chronic diseases. And lead is absorbed and distributed in the blood, bone and soft tissue (3). Blood lead levels were measured as an indicator of exposure and toxicity, and studies have shown that lead has acute effects on blood pressure through recent doses and chronic effects on the risk of hypertension through cumulative doses (4). Other studies have found that high blood pressure is associated with high blood lead levels in the west of Scotland, which may explain the high incidence of cardiovascular disease in the area (5). Similar cardiovascular complications have been observed after excessive lead exposure in laboratory animals (6). Several candidates have been identified for the mechanisms of lead-induced hypertension, including oxidative stress, inflammation, dysregulation of vasoactive hormones, impaired nitric oxide (NO) systems, and altered cellular Ca^2+^ transport and intracellular Ca^2+^ distribution (7, 8).

In various models, and even in human studies, HDL has been shown to have multiple properties that can reasonably provide cardiovascular protection (9). It has many beneficial effects, including reverse transport of cholesterol, antioxidant, anti-inflammatory, anti-apoptotic, and as a vasodilator (10). Many studies have indicated that HDL can protect endothelial cells (11), Specifically, HDL exerts its vasodilator effect by activating (phosphorylating) the endothelial nitric oxide synthase, which increases the level of NO, the most active vasodilator in the body (12). And studies have found that blood lead has a certain influence on HDL production (13). However, clinical studies on the effect of HDL on the relationship between blood lead and hypertension risk are limited. So we hypothesized that blood lead and HDL interact with the risk of hypertension. We aimed to explore the relationship between blood lead and blood pressure and the effect of HDL on this relationship.

## Methods

### Data Selection

We used data collected by the NHANES conducted by the U.S. National Center for Health Statistics to assess the health and nutrition status of a representative sample of the non-institutionalized U.S. civilian population. All procedures in the NHANES survey cycle used in this study were approved by the Ethics Review Board of the National Center for Health Statistics Research and informed consent was given by all participants.

We included adult participants aged 20–60 years who underwent blood lead and blood pressure and HDL measurements in NHANES from 2005 to 2014. Participants with missing data on blood lead concentration, blood HDL concentration, blood pressure, and covariates were excluded, and the final study population comprised 16,451 participants.

### Measurement of Blood Pressure

BP was measured by trained inspectors using standardized protocols, and three consecutive blood pressure readings were taken after sitting quietly for 5 min and determining the maximum inflation level (MIL) of the participant. If the blood pressure measurement was interrupted or performed completely, a fourth attempt can be made. All blood pressure measurements (systolic and diastolic) were performed at a mobile screening center (MEC) (14). We calculated the mean of the first 3 systolic and diastolic readings for further analysis.

### Blood Lead Measurement

The whole blood samples were processed, stored, and shipped to the National Center for Environmental Health and centers for Disease Control and Prevention for analysis, following a simple dilution sample preparation step, using an inductively coupled Plasma Dynamic Reaction Cell mass spectrometer (ELAN DRC II, PerkinElmer, Norwalk), blood lead content in whole blood samples was directly measured by mass spectrometry (15).

### Measurement of HDL Concentration

Serum samples were stored at appropriate freezing conditions (−30°C) and then transported to the laboratory for testing. Magnesium sulfate/dextran solution was first added to the sample, followed by reagent 2, and finally measured on Roche modular P and Roche Cobas 6000 chemical analyzers (16). Blood HDL concentration was defined as low HDL (≤ 49 mg/dl) and high HDL (>49 mg/dl).

### Covariates

Several factors could influence the outcome, age, gender (male or female), race (Mexican American, non-Hispanic Black, non-Hispanic White, other Hispanic, other race-including multi-racial), Marital status (married and unmarried), family PIR, BMI (<25, 25–29.9, ≥30 kg/m^2^), Educational level (less than high school, high school graduation, college or above), diabetes, hypertension, smoking status (never = smoked <100 cigarettes in life, former = smoked <100 cigarettes in life and smoke not at all now, now = smoked moth than 100 cigarettes in life and smoke some days or every day), Work activity (non-work activity, moderate work activity, and vigorous work activity), Alcohol consumption (yes = at least 12 alcohol drinks per year vs. no = <12 alcohol drinks per year), cardiovascular disease and albumin/urine (ug/ml) were included as covariables.

The definition criteria of diabetes are as follows: (1) doctor told you to have diabetes (2) Self-reported diabetes for a long time (3) glycohemoglobin HbA1c (%) >6.5 (4) the fasting glucose (mmol/L) ≥7.0 (5) random blood glucose (mmol/L) ≥11.1 (6) 2-h OGTT blood glucose (mmol/L) ≥11.1 (7) Use of diabetes medication or insulin (8) diabetes at birth is considered type 1 diabetes. Hypertension case definitions are based on the International Society of High Blood Pressure standards and a self-reported questionnaire. Participants were identified as hypertensive if they met the following criteria: (1) current use of hypertension medication (2) based on accurate diagnosis by the physician (3) based on blood pressure measured in real-time ≥140/90 mmHg (4) self-reported questionnaire data showing physician's prior diagnosis of hypertension and current use of medication to lower blood pressure. (5) The diagnostic criteria for hypertension by ambulatory blood pressure monitoring (ABPM) were: mean blood pressure ≥130/80 mmHg within 24 h, daytime ≥135/85 mmHg, at night ≥120/70 mmhg. In addition, CVD is determined by any reported diagnosis of congestive heart failure, coronary heart disease, angina, heart attack, or stroke (17). Residents were asked “Has a doctor or other health professional ever told you that you have congestive heart failure/coronary heart disease/angina/heart attack/stroke?” and participants who answered “yes” to either question were included in our study's general cardiovascular disease group.

### Statistical Analysis

Data analysis was performed using a statistical software package R (http://www.R-project.org, R Foundation). During statistical analysis, complex multistage stratified sampling was analyzed using appropriate stratification, clustering, and weights. Multiple linear regression analysis was used to study the relationship between blood lead and blood pressure. SBP and DBP mean values were assessed at different HDL concentrations. The likelihood ratio test was used to examine the interactions between subgroups.

In the descriptive analysis, continuous variables are represented as mean and standard deviation (SD) or median and quartile range (QR), and categorical variables as weighted percentages (%). Calculate 95% confidence intervals (Cls). The statistical significance level was set at *p* < 0.05.

The continuous variables were evaluated by chi-square test, *T*-test (normal distribution), and Kruskal-Wallis test (skewness distribution).

## Results

### Baseline Characteristics of the Study Population

**Figure 1:**
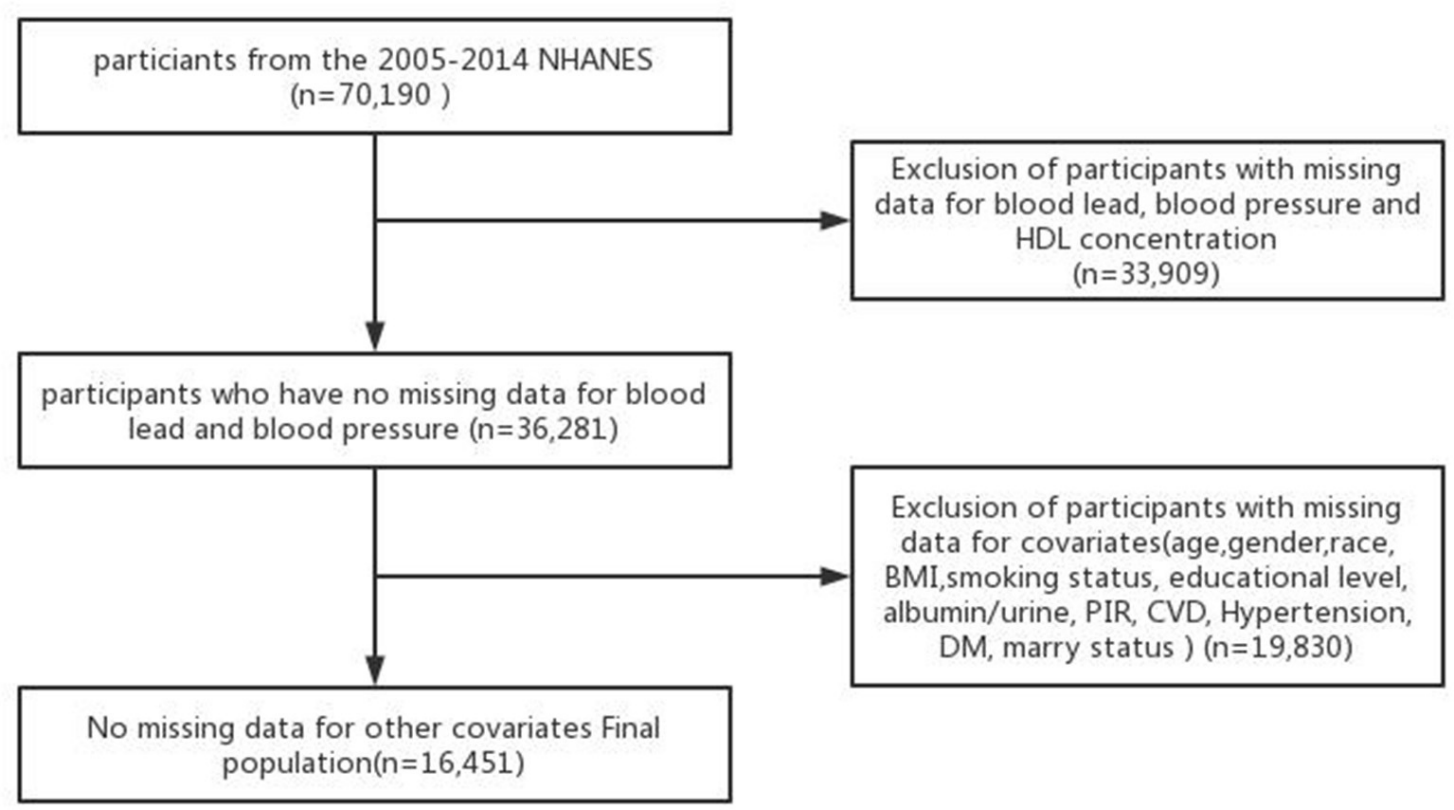
Flowchart for exclusion criteria.

Six NHANES cycles (2005–2006, 2006–2007, 2007–2008, 2009–2010, 2011–2012, and 2013–2014) were used in this study. After several screenings to exclude participants with missing covariate data, the remaining 16,451 participants were included in our analysis. A flowchart for exclusion criteria is shown in Figure 1.

Table 1 shows the study population according to the high and low blood concentrations of HDL (HDL ≤ 49 mg/dl for low concentration, HDL > 49 mg/dl for high concentration). Compared with low HDL concentration, people with high HDL concentration may be female, non-Hispanic White, married, higher PIR, lower BMI, well-educated, no diabetes, no hypertension, non-work activity, no cardiovascular disease, no smoking, and low albumin/urine. No statistically significant differences were detected in age and alcohol use (all *p*-values were >0.05).

**Table 1:** Baseline characteristics of the study population.

	**High density lipoprotein (mg/dl)**			
**Variables**	**Total (*n* = 16,451)**	**HDL ≤49 (mg/dl)** **(*n* = 8,015)**	**HDL > 49 (mg/dl)** **(*n* = 8,436)**	***p***
Age, Median (IQR)	40.0 (30.0, 50.0)	40.0 (30.0, 50.0)	40.0 (29.0, 50.0)	0.359
Gender, *n* (%)				<0.001
Female	8,472 (51.5)	2,964 (37)	5,508 (65.3)	
Male	7,979 (48.5)	5,051 (63)	2,928 (34.7)	
Race, *n* (%)				<0.001
Mexican American	2,812 (17.1)	1,597 (19.9)	1,215 (14.4)	
Non—hispanic black	3,388 (20.6)	1,352 (16.9)	2,036 (24.1)	
Non—hispanic white	6,769 (41.1)	3,342 (41.7)	3,427 (40.6)	
Other hispanic	1,541 (9.4)	818 (10.2)	723 (8.6)	
Other race- including multi-racial	1,941 (11.8)	906 (11.3)	1,035 (12.3)	
Marital status, *n* (%)				<0.001
No	3,826 (23.3)	1,724 (21.5)	2,102 (24.9)	
Yes	12,625 (76.7)	6,291 (78.5)	6,334 (75.1)	
PIR, Mean ± SD	2.5 ± 1.7	2.4 ± 1.6	2.7 ± 1.7	<0.001
BMI, *n* (%)				<0.001
<25	4,975 (30.2)	1,420 (17.7)	3,555 (42.1)	
25–29.9	5,327 (32.4)	2,720 (33.9)	2,607 (30.9)	
≥30	6,149 (37.4)	3,875 (48.3)	2,274 (27)	
Educational level, *n* (%)				<0.001
Less than high school	3,548 (21.6)	1,961 (24.5)	1,587 (18.8)	
High school graduation	3,676 (22.3)	1,963 (24.5)	1,713 (20.3)	
College or above	9,227 (56.1)	4,091 (51)	5,136 (60.9)	
DM, *n* (%)				<0.001
No	13,445 (81.7)	6,146 (76.7)	7,299 (86.5)	
Yes	3,006 (18.3)	1,869 (23.3)	1,137 (13.5)	
Hypertension, *n* (%)				<0.001
No	11,993 (72.9)	5,545 (69.2)	6,448 (76.4)	
Yes	4,458 (27.1)	2,470 (30.8)	1,988 (23.6)	
Marital status, *n* (%)				<0.001
Never	9,451 (57.4)	4,311 (53.8)	5,140 (60.9)	
Former	2,967 (18.0)	1,500 (18.7)	1,467 (17.4)	
Now	4,033 (24.5)	2,204 (27.5)	1,829 (21.7)	
Work activity, *n* (%)				<0.001
Non-work activity	7,708 (46.9)	3,669 (45.8)	4,039 (47.9)	
Moderate work activity	3,224 (19.6)	1,558 (19.4)	1,666 (19.7)	
Vigorous work activity	3,348 (20.4)	1,862 (23.2)	1,486 (17.6)	
Unknown	2,171 (13.2)	926 (11.6)	1,245 (14.8)	
Alcohol, *n* (%)				0.388
Yes	3,113 (18.9)	1,524 (19)	1,589 (18.8)	
No	9,950 (60.5)	4,876 (60.8)	5,074 (60.1)	
Unknown	3,388 (20.6)	1,615 (20.1)	1,773 (21)	
CVD, *n* (%)				<0.001
No	15,697 (95.7)	7,560 (94.7)	8,137 (96.6)	
Yes	706 (4.3)	423 (5.3)	283 (3.4)	
Albumin/urine ug/ml., Median (IQR)	7.2 (3.9, 14.1)	7.9 (4.3, 15.4)	6.8 (3.5, 12.9)	<0.001
lead, Median (IQR)	1.0 (0.6, 1.6)	1.0 (0.7, 1.6)	1.0 (0.6, 1.6)	<0.001

### The Connection Between Blood Lead and Blood Pressure

In DBP, in the second model, β was negative, but by model 5, β was positive and the *p*-value had been > 0.05 (Table 2). It is not statistically significant, so the relationship between blood lead obtained in DBP and blood pressure is not reliable. In contrast, in SBP, fully adjusted β > 0, *p* change little and both <0.05, so the Connection between blood lead obtained in SBP and blood pressure is reliable, and blood lead is positively correlated with blood pressure (β: 0.35, 95 Cl: 0.16–0.55).

**Table 2:** Association between blood lead and blood pressure.

**Models**	***n***	**DBP**		**SBP**	
		**β_95 Cl**	***P*** **_value**	**β_95 Cl**	***P*** **_value**
Model1	16,451	0.8 (0.62–0.97)	<0.001	1.8 (1.57–2.02)	<0.001
Model2	16,451	−0.16 (−0.33–0.01)	0.066	0.25 (0.03–0.46)	0.026
Model3	16,451	0.01 (−0.17–0.18)	0.93	0.33 (0.12–0.55)	0.003
Model4	16,451	0.12 (−0.05–0.29)	0.157	0.37 (0.17–0.57)	<0.001
Model5	16,451	0.11 (−0.06–0.28)	0.186	0.35 (0.16–0.55)	<0.001

*Model1: not adjusted*.

*Model2: adjusted for age, gender, and race*.

*Model3: model2+ Marital status, BMI, PIR, and education level*.

*Model4: model3+DM, Hypertension, smoking status, work activity, alcohol consumption, and CVD*.

*Model5: model4+ albumin/urine*.

*DBP, diastolic blood pressure; SBP, systolic blood pressure; PIR, poverty income ratio; BMI, body mass index; DM, diabetes mellitus*.

### Effect of HDL Concentration and Blood Lead on SBP

In model 1, we did not add any covariates to control confounding factors. At this time, HDL ≤ 49 (mg / dl) group, β = 0.94 and *p* < 0.001; HDL > 49 (mg / dl) group, β = 2.95 and *p* < 0.001. In model 2, we adjusted three confounding factors: age, gender, and race, which belong to an important part of population characteristics. At this time, HDL ≤ 49 (mg / dl) group, β = 0.06 and *p* = 0.661; HDL > 49 (mg / dl) group, β = 0.55 and *p* = 0.002 (*p* < 0.05). The results show that in HDL ≤ 49 (mg / dl) group, the effect value β has become unstable, with *P* > 0.05, which is not statistically significant. In model 3, we continued to adjust the remaining socio-economic demographic characteristics and BMI: marital status, BMI, PIR, education level at that time, HDL ≤ 49 (mg/dl) group, β = 0.13, *p* = 0.347; HDL > 49 (mg / dl) group, β = 0.57, *p* = 0.001 (*p* < 0.05). In the low HDL group, the *p*-value was still not statistically significant. In model 4 and model 5, we continued to adjust some diseases and risk or protective factors that may lead to vascular diseases and changes in blood pressure. The final result: HDL ≤ 49 (mg / dl) group, β = 0.21 and *p* = 0.109; HDL > 49 (mg / dl) group, β = 0.47 and *p* = 0.004 (*p* < 0.05). In the high HDL group, the *p*-value of β remained stable, while in the low HDL group, most of the *P*-values of β are not statistically significant (Table 3).

**Table 3:** Interactive effect of blood lead and HDL on SBP.

**Models**	**HDL ≤49 (mg/dl)** **(*n* = 8,015)**		**HDL > 49 (mg/dl)** **(*n* = 8,436)**		***p*** **for interaction**
	**β_95 Cl**	***P*** **_value**	**β_95 Cl**	***P*** **_value**	
Model1	0.94 (0.65–1.23)	<0.001	2.95 (2.6–3.31)	<0.001	<0.001
Model2	0.06 (-0.22–0.34)	0.661	0.55 (0.2–0.89)	0.002	<0.001
Model3	0.13 (-0.14–0.41)	0.347	0.57 (0.23–0.92)	0.001	<0.001
Model4	0.23 (-0.02–0.49)	0.073	0.48 (0.16–0.8)	0.003	<0.001
Model5	0.21 (-0.05–0.46)	0.109	0.47 (0.15–0.79)	0.004	<0.001

*Model1: not adjusted*.

*Model2: adjusted for age, gender, and race*.

*Model3: model2+marry status, BMI, PIR, and education level*.

*Model4: model3+DM, Hypertension, smoking status, work activity, alcohol consumption, and CVD*.

*Model5: model4+ albumin/urine*.

*HDL, high-density lipoprotein; DBP, diastolic blood pressure; SBP, systolic blood pressure; PIR, poverty income ratio; BMI, body mass index; DM, diabetes mellitus*.

It can be clearly seen that in low concentrations of HDL (HDL ≤ 49 mg/dl), β varied from negative to positive, with a large range of data, *p* was variable, and most of *p* was >0.05, not statistically significant. In high concentrations of HDL (HDL > 49 mg/dl), the β values of the five models were positive, and the changes of *p* were small and all *P* was <0.05, which was statistically significant, indicating that when HDL was high, the relationship between blood lead and HDL on SBP was reliable and showed a positive correlation. In the fully adjusted model (Model 5), there was a significant interaction between high HDL concentration and blood lead and SBP (*P*-value of interaction likelihood ratio test < 0.05).

## Discussion

In our study, both blood lead and high HDL concentration were significantly correlated with SBP. After adjustment, the association remained significant. In NHANES, the sample size is large and the level can represent the general population. And the measurement of these data was carried out with strict quality control and strict protocol. Therefore, the relationship we obtained among blood lead, HDL, and blood pressure was applied to the whole population.

In some previous studies, a link has been found among lead exposure and subsequent hypertension (HTN) and cardiovascular disease. Lead can induce hypertension in rats, and lead acts on many parts of the cardiovascular system, possibly affecting blood pressure through the renin-angiotensin system (18). A large number of animal experiments showed that long-term exposure to low concentration lead can lead to arterial hypertension, but explain low long-term environmental lead exposure to the influence of high blood pressure, the exact mechanism is unclear, underlying mechanisms including increased oxidative stress, the stimulation of renin-angiotensin system, as well as cut and nitric oxide cyclase guanylic acid (19). In humans, lead toxicity has been associated with increased cardiovascular risk and may be associated with impaired antioxidant metabolism and oxidative stress, but the shape of the dose-response relationship still needs to be explored (20, 21).

Potential mechanisms of HDL action in humans include stimulation of reverse cholesterol transport, antioxidant and anti-inflammatory properties, reduction of endothelial dysfunction, anticoagulation, and prostacyclin half-life (22). HDL has classically been thought to have a protective effect on atherosclerosis because of an inverse relationship between HDL-C concentration and coronary artery disease: each 1 mg/dL increase in HDL-C reduces the risk of cardiovascular disease by 2–3% (23). However, new data cast doubt on this, for example, HDL-elevating drugs do not reduce CV risk (24). This is due to the fact that only 5% of HDL-C comes from macrophage cholesterol efflux, and HDL-C does not represent many important anti-atherosclerotic HDL properties (for example, anti-inflammatory or vascular relaxants) (10). Therefore, HDL-C may be an insensitive method to quantify the anti-atherosclerosis properties of HDL. This is why HDL function is considered more important than HDL-C levels (25). In fact, HDL function and cholesterol outflow predict CAD events while HDL-C does not (26), confirming the fact that HDL function (quality) is more important than HDL-C level (quantity).

S1P is a bioactive lysophospholipid that is derived from the ubiquitous membrane lipid sphingomyelin. Only S1P-bound HDL, which accounts for 60% of total plasma S1P, has functional activity. Apolipoprotein (apo) M has been identified as a S1P-binding protein in HDL. S1P is the main substance responsible for the vasodilatory properties of HDL (27). The level of HDL-bound S1P in patients with CHD was lower than that in healthy volunteers, and the concentration of HDL-bound S1P was negatively correlated with the severity of CHD (28). The results of *in vitro* experiments explain the protective effect of S1P on atherosclerosis. S1P has the vasodilation effect of HIGH-DENSITY lipoprotein because S1P activates endothelial nitric oxide synthase, stimulates endothelial nitric oxide release, and induces vasodilation (27). S1P also shows endothelial protection because HDL-bound S1P enhances endothelial cell survival and migration (29).

Studies have shown that S1P improves HDL function (30). HDL-induced endothelial signaling is mediated by the S1P load because it is completely eliminated in the presence of S1P receptor antagonists and S1P neutralizing antibodies. Most importantly, S1P-loaded HDL particles significantly improved CAD-HDL function, as demonstrated by HDL-mediated signal transduction and enhanced HDL vasodilation *in vitro*.

However, some studies have found a relationship between blood lead and HDL. Women with MetS had higher HDL, and although blood lead in both groups was below the threshold range, blood lead in the experimental group (MetS group) was lower (31), the population had low blood lead and high HDL concentration. HDL concentrations were significantly higher in occupations with less lead exposure, suggesting that lead may block HDL production, but the initial dose is not clear, or the reduced effect of lead on HDL may be at higher doses (13). However, some researchers have found that with the increase of blood lead level, HDL also increases significantly, which may be related to age, gender, and sample size (32).

These correlations can demonstrate and explain the interaction effect of blood lead and HDL concentration on blood pressure to a certain extent. It makes sense that lead at low HDL-C levels can cause hypertension, but high HDL-C levels can reduce the hypertensive effect of lead through THE S1P carried by HDL particles. These studies are still very limited, but we can be a director of the research in the future, lead to high blood pressure of the threshold value has not been fully elucidated, with blood lead levels and high levels of HDL may lead to high blood pressure, the findings of the study helps to increase blood lead levels and high levels of HDL alert, and may have significance for clinical prevention and treatment. Of course, this aspect also needs more research to demonstrate.

This study still has limitations, which are reflected in the defects of the cross-sectional study itself, the deviation of the return visit, the accuracy of the questionnaire answer collection, confounding factors affecting the experimental data, possible errors in the measured samples, and even errors in the database. If there are different articles with different results, the results may be different due to different covariables considered or different database use, and different characteristics such as the age of the selected population, which will lead to different results. In addition, we did not assess HDL function, and we cannot prove whether lead causes HDL dysfunction; We did not measure S1P levels in HDL particles. It is easy to speculate that lead reduces S1P in HDL and thus reduces HDL function (33). There are several metals that can affect HDL and cause atherosclerosis, such as cadmium (34). Many patients have elevated levels of several metals at once, not just lead. Thus, HDL may be affected by lead, but it may also be affected by other metals such as cadmium.

## Conclusion

In general, through this study, we found that blood lead and high HDL concentration together affect SBP. While we have provided some clinical clues, further research is needed to provide more evidence.

## Data Availability Statement

NHANES has developed a public use dataset, available at: https://www.cdc.gov/nchs/nhanes/index.htm. Users can download relevant data for free for research and publish relevant articles. Our study is based on open source data, so there are no ethical issues and other conflicts of interest.

## Ethics Statement

The authors are responsible for all aspects of the work to ensure that issues related to the accuracy or completeness of any part of the work are properly investigated and resolved. This research was conducted following the Declaration of Helsinki (revised 2013).

## Author Contributions

BH and P-yH: conception and design. N-nZ, Z-mG, and J-tF: collection and assembly of data. J-lG: drafting the work or revising it critically for important intellectual content. C-qH: substantial contributions to the conception or design of the work. All authors: data analysis and interpretation, manuscript writing, and final approval of manuscript.

## Funding

This study was supported by grant from Zhongnanshan Medical Foundation of Guangdong Province (ZNSA-2020013) and grant from Guangzhou Municipal Science and Technology Project (202102010168).

## Conflict of Interest

The authors declare that the research was conducted in the absence of any commercial or financial relationships that could be construed as a potential conflict of interest.

## Publisher's Note

All claims expressed in this article are solely those of the authors and do not necessarily represent those of their affiliated organizations, or those of the publisher, the editors and the reviewers. Any product that may be evaluated in this article, or claim that may be made by its manufacturer, is not guaranteed or endorsed by the publisher.
